# Detection and characterisation of bovine rotavirus in Ireland from 2006–2008

**DOI:** 10.1186/2046-0481-67-13

**Published:** 2014-06-20

**Authors:** PJ Collins, Emily Mulherin, Olivia Cashman, Grainne Lennon, Lynda Gunn, Helen O’Shea, Séamus Fanning

**Affiliations:** 1 Department of Biological Sciences, Cork Institute of Technology, Rossa Avenue, Bishopstown, Cork, Ireland; 2 UCD-Centre for Food Safety, School of Public Health, Physiotherapy & Population Science, University College Dublin, Belfield, Dublin 4, Ireland; 3 Herd and Veterinary Public Health Unit, School of Veterinary Medicine, University College Dublin, Belfield, Dublin 4, Ireland

**Keywords:** Bovine rotavirus, Epidemiology, Genotyping, Vaccine

## Abstract

**Background:**

Worldwide, Group A bovine rotavirus (RVA boRV) is one of the main causes of neonatal calf diarrhoea. Currently, limited epidemiological and sequence data exists on the RVA disease in bovines in Southern Ireland only. The aim of the study was to generate epidemiological and sequence data of RVA boRV distributed over a wide geographical area in Ireland.

**Findings:**

272 stool samples were obtained from symptomatic calves and analysed to identify the prevalent G and P genotypes. Viral type combinations including G6P[5], G6P[11] and G10P[11] genotype were the most frequently identified. The G6P[5] combination was predominant throughtout the study, accounting for 70% (n = 191). Sequence analysis of the VP7 gene revealed that Irish G6 strains fell within Lineage IV, similiar to previous reports in Ireland.

**Conclusion:**

The detection of unusual G and P combinations may have an impact on rotavirus control programmes and current vaccines may need to incorporate new strains, as the current vaccine available may not offer protection against all of these circulating types.

## Findings

Group A Rotavirus (RVA) belongs to the family *Reoviridae,* and consists of 11 segments of double-stranded RNA (dsRNA), encoding six structural viral proteins (VP1–4, VP6–7) and six non-structural proteins (NSP1–6), enclosed in a triple layered protein capsid. Rotaviruses are classified into eight antigenically or genetically distinct groups (RVA to RVH), on the basis of the common group antigen, the inner capsid protein VP6. RVA, RVB, RVC and RVH are associated with acute gastroenteritis in humans and animals [1].

The two outer capsid proteins, VP7 and VP4, independently elicit neutralizing antibodies, induce protective immunity, and are used to classify rotaviruses into G (for glycoprotein) and P (for protease-sensitive) types, respectively [2]. Recently, a novel sequence-based classification system has been proposed, based on nucleotide identity cut-off percentages, and different genotypes have been defined for each genome segment [2].

Neonatal diarrhoea induced by RVA boRV causes significant economic loss in the dairy and beef industry due to increased morbidity and mortality, treatment costs, and reduced growth rates [3]. G6, G8, G10 are the most predominant G types in bovines P[1], P[5] and P[11] have been recorded in the majority of bovine diarrhoeal cases [4,5]. Previous studies in Ireland have indicated that genotypes G6 and G10 in various combinations with P genotypes P[1], P[5] and P[11] were the only circulating genotypes in the Irish bovine population [6,7].

In Ireland, limited data exists on the epidemiology of RVA disease in bovines. Molecular and sequence analysis incidence of RVA in bovines has only been investigated since 2002. These studies were limited to herds from only one region in Southern Ireland [6,7]. In the present study, samples were received from collaborating Dept of Agriculture Fisheries and Food Regional Veterinary Laboratories (DAFF RVLs), distributed over a wide geographical area in Ireland.

## Methods

A total of 272 feacal samples positive for bovine RVAs (collected from 2006–2008) were obtained from collaborating RVL located in Athlone, Kilkenny, Limerick, and Sligo. Total nucleic acids were extracted from the samples by a standard phenol-chloroform method with ethanol precipitation. The extracted nucleic acids were re-suspended in 100 μl of sterile DEPC H20 and stored at -80°C prior to use.RT-PCR and G and P typing assays for bovine rotavirus samples were conducted using methods as previously described [6,7]. The amplicons were analyzed in 1.5% agarose gels following ethidium–bromide staining and UV light transillumination.

Following RT-PCR, the VP7-encoding genes of five Irish bovine rotavirus strains were directly ligated to pCR2.1 (Invitrogen, Bv, Amsterdam, The Netherlands) and cloned according to manufacturers’ instructions. The constructs were screened for the correct inserts prior to purification with the Wizard SV Gel and PCR clean up system (Promega). These constructs were commercially sequenced (Qiagen Genomic Services, Qiagen Ltd., Germany) using M13 forward and reverse sequencing primers. Phylogenetic and molecular evolutionary analyses were conducted with MEGA version 2.1 (Arizona State University, USA) [8]. GenBank accession numbers GQ433984, GQ433985, GQ433986 and GQ433987 were assigned to UCD/RVL-Bov 1, UCD/RVL-Bov 2, UCD/RVL-Bov 3 and UCD/RVL-Bov 4 respectively.

## Results

Previous studies in Ireland have indicated that combinations of G and P types have varied from 2002–2009 [6,7]. Overall, the results observed in the current study indicated that genotype G6 (87%) was the dominant G-type (data not shown). Genotype G10 was identified in 10% of the collection. Generally, the levels of G6 and G10 in this study are in agreement with previous studies in Ireland [6,7]. In previous studies, P[5] was detected at fluctuating levels from 40.9-77.8% [6,7]. P[5] was the dominant P genotype in the current study and was detected at a rate of 65% (data not shown).

**Figure 1:**
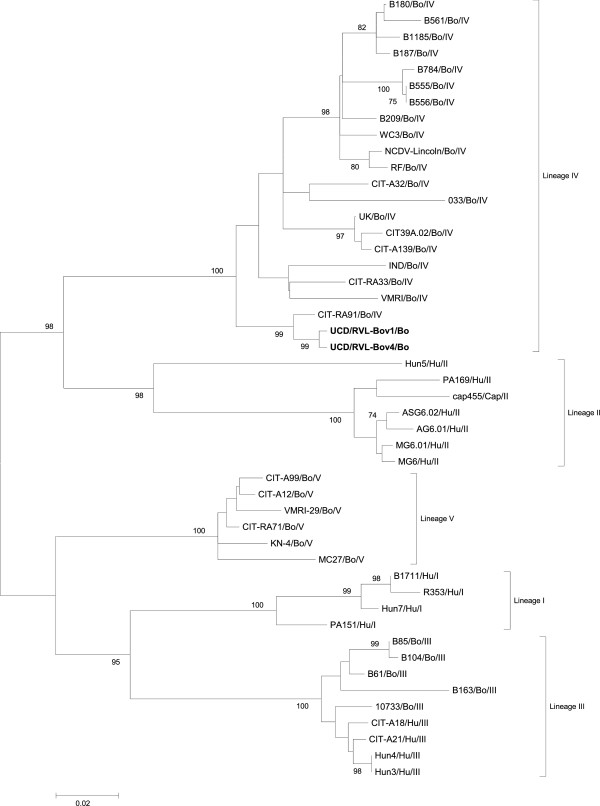
**Phylogenetic tree based on the VP7 nucleotide sequences of a selection of bovine and human G6 strains with bovine strains UCD/RVL-Bov 1 and UCD/RVL-Bov 4.** The Irish bovine strains are highlighted in bold.

The G- and P-genotypes identified were distributed in nine binary combinations (Table 1). The most frequent G- and P-type association was G6P[5], accounting for 70% (n = 191) of the isolates examined and 6.6% (n = 18) were G10P[11], both considered the most common G- and P-type combinations found in bovines [4,5]. Other combinations were also detected including G6P[11] (8%, n = 22), G10P[5] (0.7%, n = 2) and G6P[1] (1.4%, n = 4). The most common mixed infection detected was G6P[5 + 11] (7.7%, n = 21), while G6G10P[11] (2.5%, n = 7), G6G10P[5] (1.4%, n = 4) and G6P[1 + 11] (1.1%, n = 3) were also detected (Table 1). A phylogenetic tree, based on a selection of human and bovine GARV G6 was constructed (Figure 1). The Irish strains UCD/Bov/RVL-01 and UCD/Bov/RVL-04 clustered with G6 strains in lineage IV. Lineage IV contains a number of Irish strains identified in previous studies [6] and the vaccinal strain, UK.

**Table 1:** Changing profile of circulating boRV strain types in Ireland (2002–2008)

**Combined G[P] types**	**Surveillance study period *2002-2009***^***a ***^ ***2006-2008***^***b***^	
	**n (%) of strain types**	
G6P[5]	123(37.0)	191 (70.2)
G10P[11]	42 (12.7)	18 (6.6)
G6P[1]	3 (0.9)	4 (1.4)
G6P[11]	37 (11.1)	22 (8.0)
G10P[5]	8 (2.4)	2 (0.7)
G6P[5+11]	83 (25)	21 (7.7)
G6G10P[11]	16 (4.8)	7 (2.5)
G6G10P[5]	8 (2.4)	4 (1.5)
G6G10P[5+11]	9 (2.7)	0
G6P[1+11]	0	3 (1.1)
Total	332	272

^a^Cashman et al., 2010, ^b^Present Study.

## Discussion

Viral type combinations including G6P[5], G6P[11] and G10P[11] genotype were the most frequently identified in this study (Table 1). Previous studies have demonstrated the presence of G6, G10, P [5], and P[11] as the main bovine rotavirus types circulating in southern Ireland (Table 1) [6,7]. Similar data was reported previously from studies across Europe where genotypes G6 and G10 combined with P[5] and P[11] were the main boRV genotype detected [4,5,9,10].

However, temporal fluctuations in the predominant G and P types have been observed in humans and other animal species [11-13]. Notably, genotype G6P[5] was found to be circulating at a much higher level (70%) in this study than previous studies conducted in southern Ireland [6,7]. These fluctuations in G and P type suggest the need for continued surveillance of the circulating types in order to determine the profiles of bovine rotavirus genotypes in the Irish beef and dairy herds.

Neonatal enteritis continues to be the most common cause of deaths in calves aged less than one month of age in the Republic of Ireland. Rotavirus, at 33.1%, was the most frequently identified pathogen in 2009 [14]. In Ireland, a vaccine directed against the G6P[5] UK rotavirus strain is licensed for use and is administered to pregnant cows to provide passive immunity to newborn calves. Calves become protected when they receive high titres of neutralising antibodies against RVA through the colostrum from the dam.

In a recent study in France [5] a bovine RVA G6P[5] vaccine did not seem to promote the emergence of rotavirus strains with genotypes or variants different from those of the vaccine. In addition, this vaccine did not seem to promote the emergence of other viruses, such as caliciviruses, which also cause calf diarrhoea.

## Conclusion

Results from this study (and previous studies) in Ireland have indicated that the current vaccine is appropriate to provide protection against RVA infection in bovines. However, as bovine RVA can undergo natural temporal fluctuations in G and P types, it may be appropriate to incorporate G10 and P[5] and P[11] into future vaccines to maintain maximum vaccine efficacy.

## Competing interests

None of the authors of this paper has a financial or personal relationship with other people or organisations that could inappropriately influence or bias the content of the paper. The authors declare that they have no competing interests.

## Authors’ contributions

PC and EM carried out the analysis and drafted the manuscript. OC, GL, LG, HOS and SF helped draft the manuscript and gave final approval of the version to be published. All authors read and approved the final manuscript.
